# The Restrospective View of Dentistry

**Published:** 1880-11

**Authors:** W. F. Fowler


### THE RETROSPECTIVE VIEW OF DENTISTRY.

READ BY W. F. FOWLER, D.D.S., BEFORE THE EAST TENNESSEE DENTAL ASSOCIATION.

The great and momentous question, which seems to agitate the minds of the celebrities of our profession at this day and time is one of wonder and astonishment.

Eclecticism at one time in the practice of medicine seemed to heap anathemas upon the advocates of the same; but the extreme measures inculcated in the practice of dentistry by such leaders as Flagg, Chase and others are, beyond a doubt, the greatest surprise of anything which could have occurred in the practice of our liberal profession. It is to me equal to the other extreme wherein gold is the only filling by which a tooth can be saved. We are fully convinced that no one material can be universally used even in individual cavities of the same class of teeth.

The density of the tooth, the proximity of the same to other teeth, the condition of the oral cavity, all must be considered when these practical remedies are at stake.

We are frank to confess that in our belief, gold in many instances is far inferior to a plastic filling, taking into consideration the preservation of the natural organs. But it is an uncontrovertable fact, that to arrive at any degree of distinction an individual must have at *heart* that object for which he aspires.

The end for which we should look as dentists, is the restoration of that organ to permanency and to color, as well as to its condition, and to do this requires not only manual skill of the highest order but a theoretical knowledge, to know how and when to strike, what filling is best suited to each cavity. This we say is the desideratum of which very many are deficient.

We find that the dollar is the least to be considered as far as the object to be obtained is concerned, both as to patient and operator. We do not propose to set ourselves up as a target, neither do we think our influence extensive, but according to usage we have a right to our opinion and shall advocate it until better convinced.

The extreme "hobbies" of this day and time, especially those emanating from such men as Flagg and Chase we must say—is deleterious to our calling and to the colleges of our land.

The young men just embarking in the dental profession are stunned, vexed and annoyed, they know not what to do, whom to follow or what to say. This we say from *sad* experience.

Look if you please at the different materials in the different journals and see if you can tell as a student what road to take across the plain of dentistry. Tell me how a young man can know how to proceed in his chosen avocation? Only we say—without a successful contradiction, by actual practice—a plain path in this day of enlightment should be clearly and definitely laid out.

Look again at the experience of many in our own beloved East Tennessee, with the "Sponge-gold" as a sample, look at the different plastic fillings as well as at the different makes and forms of gold as advocated by *us all* and tell me, brother, which is the best. You cannot, nay you will not. Then the only advice, young man, is look for yourself, judge for yourself and act accordingly. How is this for an enlightened age?

Well! what is the remedy? We would suggest that a convention should be called of all leading dentists and dealers to fully discuss this matter, without any partiality, and with honest experience, advocate that which is good only on years of trial, and reject that which is worthless. Then the student will know exactly what course to pursue and by application and practice he will be a benefactor to humanity and an honor to himself.

It is a conceded fact that all cavities must be cleansed of all foreign matter unless immediately over the pulp, that they must have a certain uniform shape and that one number of a certain make of gold is really sufficient for any tooth, that one mode of preparing the foil ready for use is really sufficient, that either the mallet or automatic-plugger, is sufficient for the introduction of the same is also true, and that the finishing is always the same.

As to plastic fillings an established make can be certainly and truly agreed upon. Thus far the course is a plain one and the oftener followed the more familiar it will become. Hence the student with these guides can pursue his course knowingly and intelligently, no necessity for a wavering either to the right or left; but according to the present status of things he knows not what to do, but is faltering between two opinions, this will be the case as long as such really exists—viz.: the extreme measures as taught by dentists of long experience and world wide fame. Who has not noticed the peculiar advantages and superiority for the different makes and forms of gold, each claiming that nothing short of theirs should be used. Some again claim that no amalgam can possibly preserve the tooth but theirs, that soft or non-cohesive foil only should be used, whilst others say certain No's. of cohesive foil alone is admissable. Again certain celebrities say "*no gold used at all*," "more than three-fourths of all gold plugs leak," another comes to the front with "Eureka," "Sponge-gold," and Pack's pellets alone, gutta percha, acme cement, oxy-chlorides of zinc, phosphates, &c. What is the young man who is seeking for knowledge to do?

Teachers in dental colleges saying no gold at all. Retrograding, yes! going back in the operative as well as the prosthetic branch. We said we were retrograding, this is true.

Twenty-five or thirty years ago one method alone was employed in introducing gold—that of hand pressure, one or two forms of gold—the rope and the ribbon with principally No's. 4 and 6 foil, now the practice is so varied, scarcely any two operate alike, hence the trouble with the student of dentistry.

Gold too was advocated and used by us who inserted dentures twenty years ago. Now it is a rare thing to see such a case, few if any indeed are prepared to even set a tooth on gold.

A *practical* knowledge seems to be the desideratum in my portion of the State without any theory whatever.

And those who have taken a thorough course and gained a practical knowledge of dentistry are cutting their own throats by imparting that instruction of an age of toil to the so-called "dentist," who cannot diagnose a case of "toothache," of an exposed pulp from a case of neuralgia.

If this was remedied, our fees would justify more labor and more skill, until then and only then, may we expect to cope with the bushwhacker, the mountebank and the quack.

Then in conclusion let us, brethren, try to remedy the evil, and bring about a better state of things in our profession.

